# Informing the pandemic response: the role of the WHO’s COVID-19 Weekly Epidemiological Update

**DOI:** 10.1136/bmjgh-2023-014466

**Published:** 2024-04-04

**Authors:** Juniorcaius Ikejezie, Alessandro Miglietta, Ingrid Hammermeister Nezu, Sandra Adele, Melissa M Higdon, Daniel Feikin, Harsh Lata, Samuel Mesfin, Friday Idoko, Kazuki Shimizu, Ayse Acma, Samuel Moro, Homa Attar Cohen, Mary Anissa Sinnathamby, James Richard Otieno, Yosef Temre, Brian Ngongheh Ajong, Bernadette Basuta Mirembe, Tondri Noe Guinko, Vaishali Sodagar, Craig Schultz, Joao Muianga, Stéphane De Barros, Aura Rocio Escobar Corado Waeber, Yeowon Jin, Anahi Rico Chinchilla, Yurie Izawa, Shagun Khare, Marcia Poole, Nyka Alexander, Silviu Ciobanu, Tshewang Dorji, Mahmoud Hassan, Masaya Kato, Tamano Matsui, Opeayo Ogundiran, Richard G Pebody, Manilay Phengxay, Ana Riviere-Cinnamond, Blanche Johanna Greene-Cramer, Emilie Peron, Brett Nicholas Archer, Lorenzo Subissi, Zyleen Alnashir Kassamali, Adedoyin Awofisayo-Okuyelu, Olivier le Polain de Waroux, Esther Hamblion, Boris Igor Pavlin, Oliver Morgan, Ibrahima Socé Fall, Maria D Van Kerkhove, Abdi Mahamud

**Affiliations:** 1 Health Emergencies Programme, World Health Organization, Geneva, Switzerland; 2 Department of International Health, Johns Hopkins Bloomberg School of Public Health, Baltimore, Maryland, USA; 3 Department of Immunization, Vaccines and Biologicals, World Health Organization, Geneva, Switzerland; 4 World Health Organization Regional Office for Europe, Copenhagen, Denmark; 5 World Health Organization Regional Office for South-East Asia, New Delhi, India; 6 World Health Organisation Regional Office for the Eastern Mediterranean, Cairo, Egypt; 7 World Health Organization Regional Office for the Western Pacific, Manila, Philippines; 8 World Health Organization Regional Office for Africa, Brazzaville, Congo; 9 World Health Organization Regional Office for the Americas, Washington, DC, USA

**Keywords:** COVID-19, Epidemiology, Public Health, SARS, Respiratory infections

## Abstract

On 31 December 2019, the Municipal Health Commission of Wuhan, China, reported a cluster of atypical pneumonia cases. On 5 January 2020, the WHO publicly released a Disease Outbreak News (DON) report, providing information about the pneumonia cases, implemented response interventions, and WHO’s risk assessment and advice on public health and social measures. Following 9 additional DON reports and 209 daily situation reports, on 17 August 2020, WHO published the first edition of the COVID-19 Weekly Epidemiological Update (WEU). On 1 September 2023, the 158th edition of the WEU was published on WHO’s website, marking its final issue. Since then, the WEU has been replaced by comprehensive global epidemiological updates on COVID-19 released every 4 weeks. During the span of its publication, the webpage that hosts the WEU and the COVID-19 Operational Updates was accessed annually over 1.4 million times on average, with visits originating from more than 100 countries. This article provides an in-depth analysis of the WEU process, from data collection to publication, focusing on the scope, technical details, main features, underlying methods, impact and limitations. We also discuss WHO’s experience in disseminating epidemiological information on the COVID-19 pandemic at the global level and provide recommendations for enhancing collaboration and information sharing to support future health emergency responses.

**Summary box**

- COVID-19 has posed an unprecedented global health crisis, demanding timely, reliable information on the pandemic’s progression to inform the public and guide decision-making.
- The WHO’s COVID-19 Weekly Epidemiological Update (WEU) provided regular, comprehensive and authoritative analyses of the global COVID-19 situation.
- The production of the WEU included several steps, which were standardised, regularly refined and automated when possible, to ensure consistency and accuracy.
- Addressing the persisting challenges inherent to the global surveillance of COVID-19, many of which were exemplified by the WEU, will require sustained international collaboration, commitment and investment.
- The methodology and lessons learnt from the experience of the WEU offer a blueprint for the development of information products that can support the response to future major health emergencies.

## Introduction

On 5 May 2023, the Director-General of the WHO announced that COVID-19 no longer constitutes a public health emergency of international concern (PHEIC),^1^ over 3 years after the PHEIC declaration made on 30 January 2020.^2^ The COVID-19 pandemic has posed an unprecedented global health crisis, with more than 770 million confirmed cases and over 6.9 million confirmed deaths being reported globally to WHO from 1 January 2020 to 1 September 2023.^3^ Given the scale of the emergency, countries worldwide had to rapidly strengthen surveillance and reporting systems to monitor the evolution, spread and impact of SARS-CoV-2, the virus that causes COVID-19. In this context, under the framework of the International Health Regulations (IHR, 2005),^4^ WHO coordinated global response efforts and continuously assisted countries to strengthen their technical capacities across all response functions.

In line with its responsibility under the IHR to disseminate information on acute public health events that constitute a PHEIC, WHO provided regular epidemiological updates on the COVID-19 pandemic to the international community since the outbreak was first identified. After the report of a cluster of atypical pneumonia cases by the Wuhan Municipal Health Commission on 31 December 2019, WHO deployed its processes for event verification and assessment, sharing detailed information about the cluster of cases with all IHR States Parties on 5 January 2020 through the WHO Event Information Site —a secure, web-based platform used by WHO to exchange information with its States Parties. On the same day, a Disease Outbreak News (DON) report was publicly released, providing information about the number of cases and their clinical status, details about the local response interventions, and WHO’s risk assessment and advice on response measures.^5^ This was followed by nine additional DON reports while transitioning to daily situation reports on 20 January 2020.^6^

As the pandemic progressed and operational demands evolved, surveillance strategies and data sharing mechanisms by the States Parties reporting cases to WHO under the IHR requirements were adapted and refined. The frequency of the situation reports also transitioned from daily to weekly, with the first edition of the COVID-19 Weekly Epidemiological Update (WEU) being published on 17 August 2020.^7^ This shift allowed for a more in-depth weekly analysis of COVID-19 epidemiological trends, leading to a better understanding of the pandemic’s dynamics and impacts. This transition also allowed for daily case and death figures to be disseminated through global and regional WHO COVID-19 Dashboards,^8^ which became more user-friendly and dynamic mechanisms to share near real-time global, regional and national updates on the pandemic with the public.

In line with WHO’s transition of COVID-19 surveillance from emergency response to long-term disease prevention and control, the last edition of the WEU was published on 1 September 2023. The WEU has since been replaced by comprehensive global epidemiological updates on COVID-19 published every 4 weeks, with WHO Regional Offices publishing epidemiological reports on a varying frequency. While quantifying the exact reach and impact of the WEU is not possible, various metrics suggest that it had a global audience and extensive influence. Between 17August 2020 and 1 September 2023, the webpage that hosted the WEU and the COVID-19 Monthly Operational Update (initially launched as a weekly publication) was visited annually over 1.4 million times on average, with visits originating from more than 100 countries.^6^ The largest share of the audience was from the WHO Region of the America (32%), followed by the European Region (28%), the South-East Asia Region (26%), the Western Pacific Region (8%) and the African region (6%). These figures likely underestimate the true scale of the readership, given that the WEU was also available through various other channels, including WHO’s Health Emergency Dashboard^9^ and the humanitarian information platform ReliefWeb.^10^

The widespread reach of the WEU came with a significant responsibility to maintain the highest standards of data integrity to avoid inaccuracies that could cause confusion or misguide policies. In this article, we describe the main features of the WEU and the process used to ensure that the information presented is precise, reliable and timely. We also discuss limitations of the WEU as well as the persisting challenges in the global surveillance of COVID-19. Finally, we provide recommendations for enhancing collaboration and information sharing to support future health emergency responses.

## Overview of the COVID-19 WEU

The WEU comprised several sections: global and regional epidemiological overviews, updates on hospitalisations and admissions to intensive care units (ICUs), analyses of SARS-CoV-2 variants, summaries of the effectiveness of COVID-19 vaccines and Special Focus pieces on key themes and emerging issues relating to managing COVID-19 throughout the pandemic. Each section presented not only the most recent data, but also retrospective data that had just become available. The limitations and caveats surrounding the reported data were also described, providing insights into their reliability and potential biases.

Between 17 August 2020 and 1 September 2023, 158 editions of the WEU were published on WHO’s website.^6^ During the course of the pandemic, the reporting interval for analysing and presenting the data in the WEU was modified to accommodate changes to the frequency of data reporting by countries to WHO. Initially, a 7-day reporting time frame was used. From January 2023, a 28-day rolling interval was employed, which helped to account for delays in data sharing and retrospective adjustments from countries alongside smoothing out weekly fluctuations in the number of cases, deaths and hospitalisations.

### Global and regional overviews

**Figure 1:**
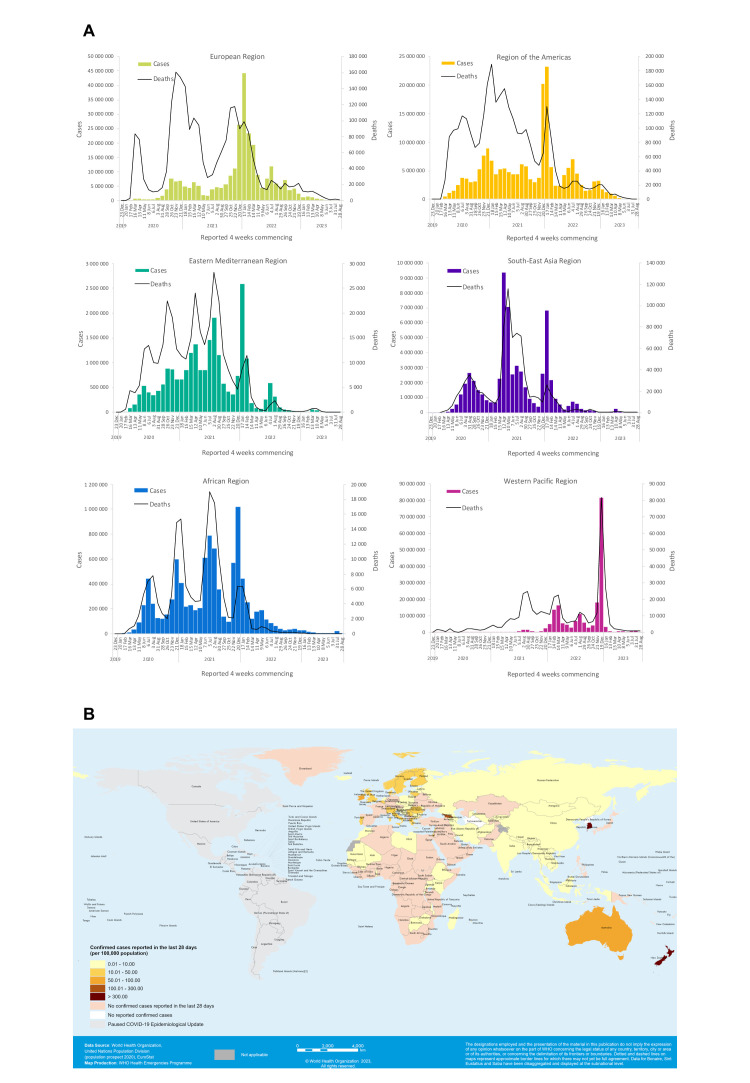
Examples of visual aids illustrating COVID-19 epidemiological trends. (A) Number of COVID-19 cases reported by WHO region and deaths by 28-day intervals, as of 1 September 2023. (B) Number of confirmed cases by country per 100 000 population, 31 July 2023–27 August 2023. Created by the WHO.^32^

Examining global and regional trends of COVID-19 cases and deaths is vital for understanding the impact of the pandemic, as well as identifying geographical areas that drive observed epidemiological trends and require enhanced public health action. In the WEU, the global and regional overviews provided descriptive analyses of the epidemiological situation both globally and by WHO region, highlighting the latest reported case and death counts, changes in epidemiological patterns over time and geographic hotspots with concerning trends. The data were presented using different visual aids, including tables, graphs and maps (figure 1).

### Hospitalisations and ICU admissions

**Figure 2:**
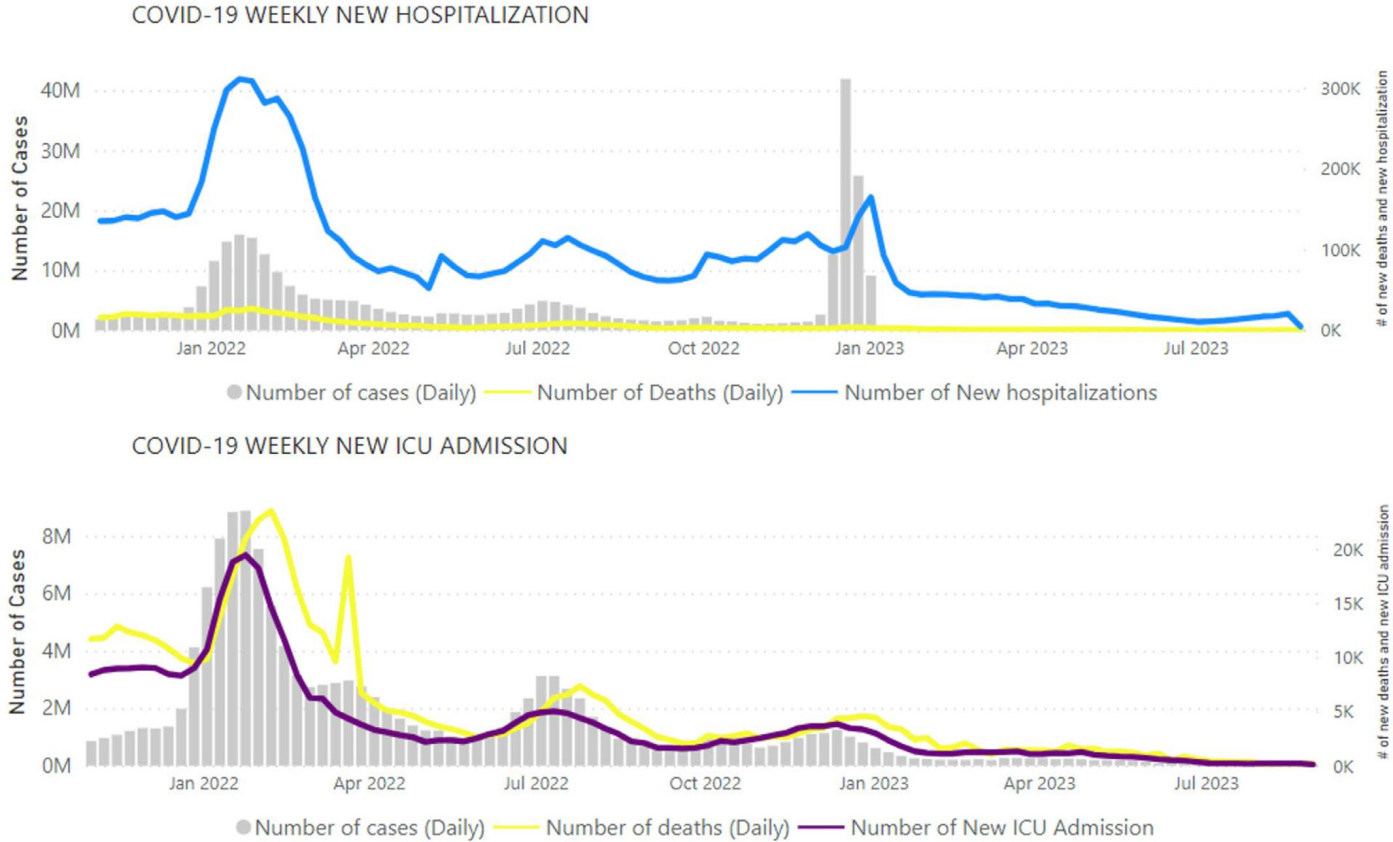
Examples of visual aids illustrating global COVID-19 cases, deaths, hospitalisations and ICU admissions reported weekly to WHO, as of 1 September 2023. The numbers of cases represented by the grey bars in the figure are only from countries reporting hospitalisations or ICU admissions, respectively. Created by the WHO.^32^ ICU, intensive care unit.

Reports on hospitalisations and ICU admissions related to COVID-19 are critical to understand the public health burden and impact of COVID-19, particularly with the emergence and spread of a several variants and need to assess their impact, as indicated in WHO’s revised surveillance guidelines^11^— increasingly so in a context of reduced community testing, which has rendered reported case counts less reliable. The morbidity section was added to the WEU in November 2022, providing an additional way to monitor epidemiological trends, track the severity of the disease, assess the pressure on healthcare systems and ultimately acquire a better understanding of the trajectory of the pandemic. The proportion of countries reporting data on these indicators was also provided for each WHO region in the text, allowing readers to assess the level of data completeness. Between 1 January 2020 and 1 September 2023, 161 countries shared data on hospitalisations at least once, and 115 countries did so for ICU admissions. During this period, reporting completeness varied significantly: the number of countries reporting data on new hospitalisations and ICU admissions ranged from 96 (41%) to 27 (12%) and 40 (17%) to 9 (4%), respectively, with a declining trend being observed since the beginning of 2022. Overall, 25 countries (11%) reported weekly new hospitalisation data at least 80% of the expected times, and 22 countries (9%) did the same for new ICU admission data. Figure 2 provides examples of visual aids used in this WEU section.

### SARS-CoV-2 variants

**Figure 3:**
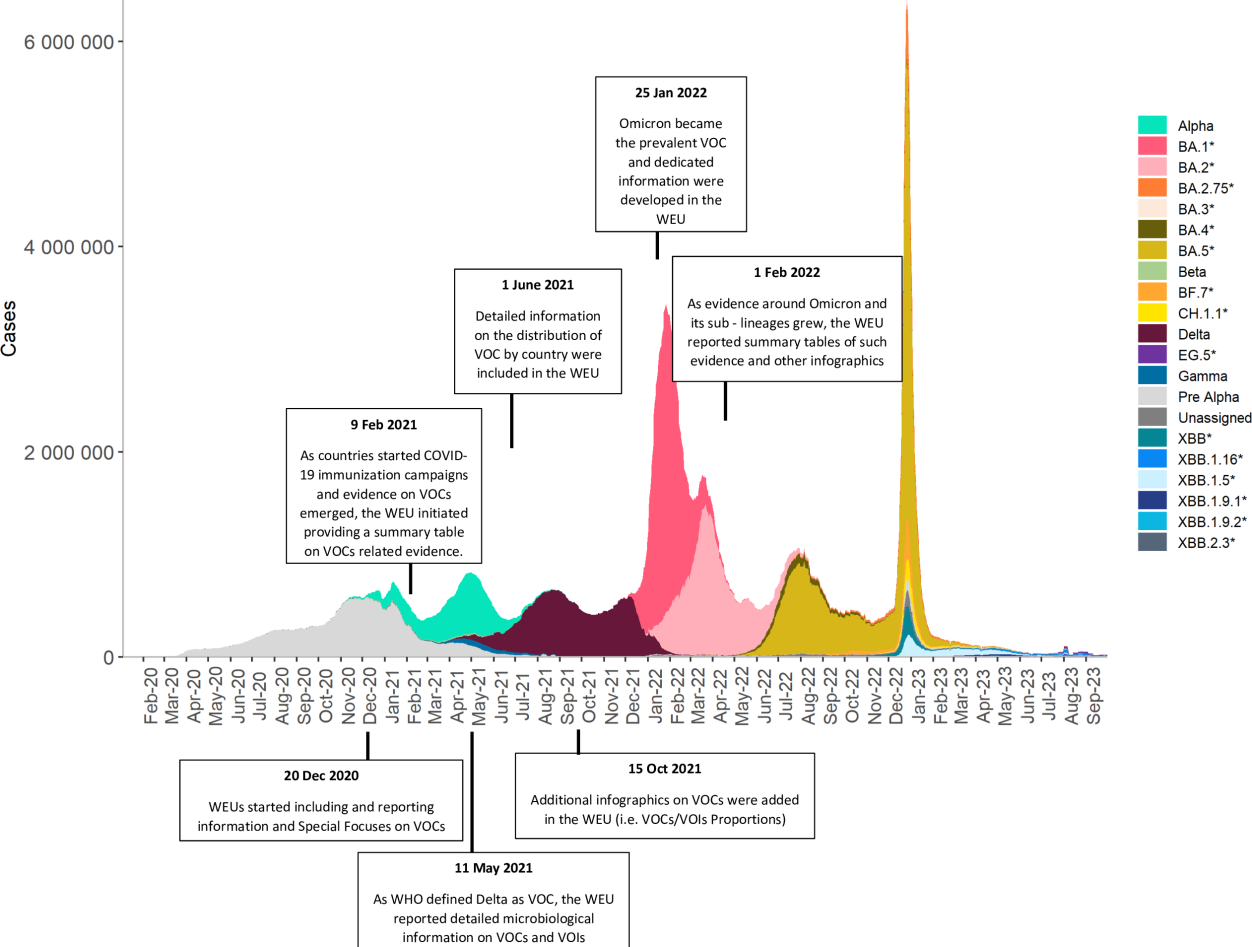
Timeline of global COVID-19 cases and dominant SARS-CoV-2 variants, 1 January 2020–1 September 2023. Created by the WHO, using data from WHO^32^ and GISAID.^14^ GISAID, Global Initiative on Sharing All Influenza Data; VOCs, variants of concern; VOIs, variants of interest; WEU, Weekly Epidemiological Update; * includes descendent lineages.

The evolution of the SARS-CoV-2 virus has involved the emergence of several variants (figure 3), some of which have resulted in significant rises in cases, hospitalisations and deaths globally. Since the start of the pandemic, there have been five SARS-CoV-2 variants of concern (VOCs): Alpha (first documented in September 2020), Beta (May 2020), Gamma (November 2020), Delta (October 2020) and the Omicron parent lineage (November 2021).^12 13^ In addition to the VOCs, there have been variants of interest (VOIs) and variants under monitoring (VUMs), which have also contributed to the overall COVID-19 burden at different times and with varying magnitudes.

The section on SARS-CoV-2 variants in the WEU was used to describe the evolution of variants, the geographical spread and prevalence of VOCs, VOIs and VUMs, alongside analysing what was known about their epidemiological and clinical characteristics. The section presented analyses on sequencing data submitted by countries to publicly available databases, such as the Global Initiative on Sharing All Influenza Data (GISAID).^14^ These analyses were fundamental for WHO and its Technical Advisory Group on SARS-CoV-2 Virus Evolution to interpret the global epidemiological trends and identifying the variants driving successive COVID-19 waves. The weekly analysis of variant data also served as a way to provide detailed information on early warning signals, drawing attention to variants with rising prevalence both regionally and globally. This section was also continuously used to communicate relevant changes and updates to the classification of variants and their associated risk assessments.

### Vaccine effectiveness

Since early 2021, with funding from the Coalition for Epidemic Preparedness Innovations, scientists at WHO, the US Centers for Disease Control and Prevention and the International Vaccine Access Center at Johns Hopkins Bloomberg School of Public Health have worked together to systematically review, evaluate and synthesise hundreds of vaccine effectiveness (VE) and neutralisation studies. The results of this living systematic review^11^ provided the content for the WEU section on COVID-19 VE.

The VE section was added to the WEU in April 2021 as results from VE studies on VOCs became available. This section synthesised the totality of the evidence, displaying the magnitude of the reduction in primary series VE against each VOC relative to the ancestral strain. Results were summarised by VOC, by vaccine and by outcome (infection, symptomatic disease and severe disease). Due to the time lags between the emergence of VOCs and the publication of VE study results about that new VOC, findings from vaccine-induced neutralising antibody studies, which are less resource-intensive and can be completed rapidly, were also reported to summarise vaccine performance as new variants emerged. For both VE and neutralisation studies, results were summarised for each vaccine with WHO emergency use listing^15^ for which data were available. In addition to the synthesis of the cumulative evidence provided in table format, an accompanying narrative provided a synopsis of the newest studies. As Delta and Omicron emerged as dominant VOCs, the focus of the synthesis and narrative shifted to the newer variants.

**Figure 4:**
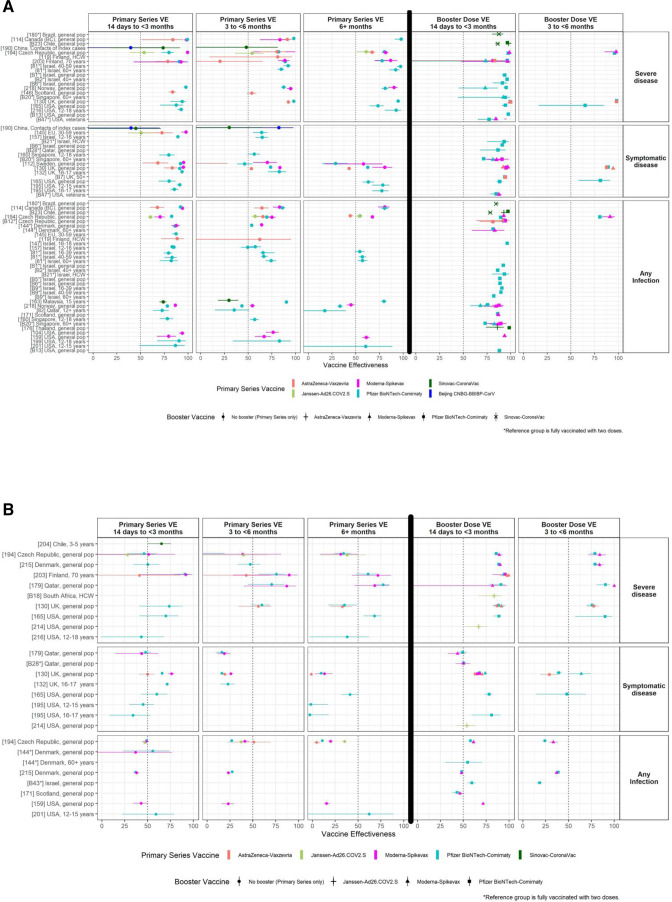
Examples of visual aids illustrating information on COVID-19 vaccine effectiveness (VE). (A) VE of primary series and booster vaccination against the Delta VOC. (B) VE of primary series and first booster vaccination against the Omicron VOC. Created by the WHO.^32^ VOC, variants of concern.

In February 2022, the VE section was updated to incorporate VE for booster vaccination and to better address questions around the duration of VE after the last dose of vaccine. This included a figure showing VE over varying time intervals postprimary and booster vaccination. For both Delta and Omicron VOCs, the accompanying figure summarised the declines in VE for individual vaccines over time for severe disease, symptomatic disease and infection (figure 4). As circulation of Delta reduced, the figures and descriptive text of the WEU focused on Omicron. In December 2022, the VE update became monthly and was simplified to a concise summary of emerging evidence against the most recent Omicron sublineages, with additional information published on VIEW-Hub—a web platform developed by the International Vaccine Access Center.^16^

### Special Focus

The Special Focus (initially called Subject in Focus) provided detailed information on specific topics relating to COVID-19. Some of the topics covered included COVID-19 in specific population groups (eg, children, adolescents, healthcare and social workers) and settings (eg, prisons, schools, residential houses) as well as response challenges in the context of the pandemic. The Special Focus also provided summaries of the periodic updates to the global rapid risk assessments, global and regional seroprevalence, overviews of newly released WHO interim guidance and guidelines (eg, for public health and social measures, isolation, contact tracing, quarantine, wastewater surveillance and mask use), policy recommendations and operational information relating to the use of COVID-19 diagnostics, therapeutics and vaccines. Although the content of some Special Focus editions on VE went on to be published in peer-reviewed journals,^17 18^ the WEU offered an opportunity to inform the international community more rapidly about these important topics. Between 17 August 2020 and 1 September 2023, 41 Special Focus editions were issued by WHO (online supplemental appendix 1).

10.1136/bmjgh-2023-014466.supp1 Supplementary data

## From data collection to WEU publication

**Figure 5:**
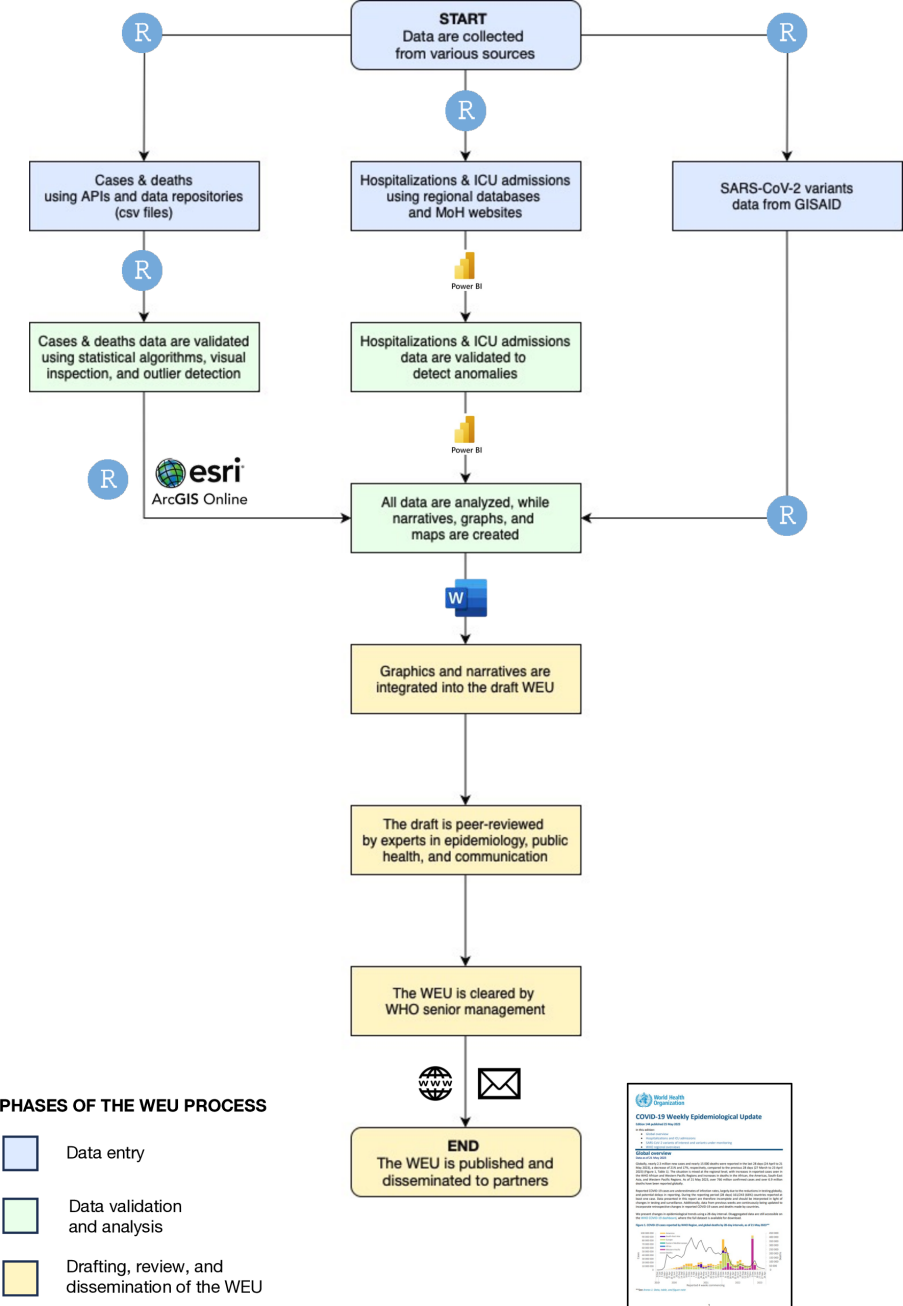
Overview of the WEU process, as of 1 September 2023. Created by the WHO. API, Application Programming Interface; GISAID, Global Initiative on Sharing All Influenza Data; ICU, intensive care unit; MoH, Ministries of Health; WEU, Weekly Epidemiological Update.

To ensure thoroughness and accuracy, the production process of the WEU was standardised and included several steps, from data entry and validation to the dissemination of the report (figure 5). Data for COVID-19 cases, deaths, hospitalisations, ICU admissions and SARS-CoV-2 variants were collected and validated on a weekly basis using multiple approaches. All data were aggregated at the national level, presenting no identifiable information.

### Data entry

Initially, data on the daily number of COVID-19 cases and deaths were sourced by WHO ROs from national health authorities through IHR channels, or directly from official national websites and/or via WHO Country Offices. In February 2023, WHO headquarters (HQ) transitioned to collecting data on a weekly basis. The data were imported by a WHO HQ data specialist on a rotational basis, using direct feeds that leveraged dedicated application programming interfaces and the R programming language,^19^ as well as via uploads (.csv files) through shared data repositories.

Data on hospitalisations and ICU admissions were collected weekly by WHO ROs as part of the detailed surveillance for COVID-19, which also included information on the age and sex of cases and deaths, number of tests conducted, test positivity rates at primary care and hospital sentinel sites, and cases and deaths among health and care workers. The data were transferred to a centralised database managed by two data specialists at WHO HQ. The data flow relied on manual entries alongside automated pipelines. These pipelines interfaced with reporting platforms specific to each WHO region, including ArcGIS Survey123, the District Health Information Software 2, the Eastern Mediterranean Flu system and The European Surveillance System. For some countries, data were collected automatically from the websites of the respective national health authorities or manually scraped from official national reports. Synergies were explored with partners (eg, European Centre for Disease Prevention and Control) to avoid duplication.

Throughout most of the pandemic, data on SARS-CoV-2 variants were collected from national health authorities. These data were supplemented by information originating from GISAID^20^ as well as other primary sources, including peer-reviewed articles, preprints and clinical trials. Variant data on SARS-CoV-2 sequences submitted by countries to GISAID were downloaded and analysed weekly using R scripts by a team of 8–10 data analysts, epidemiologists and laboratory experts to investigate global, regional and national trends. This system was also used as an early warning tool to assess rising prevalence of specific variants.

### Data validation and analysis

To ensure accuracy and consistency, collected data were validated through several approaches. For cases and deaths, a WHO HQ epidemiologist on rotation, initially examined the differences in daily and weekly changes in reported figures at the country level; then assessed epidemic curves to identify abnormal spikes in cases and deaths; and finally employed statistical algorithms to detect outliers and anomalies in the dataset, such as negative or missing values. The validation process was conducted using the Shiny app package from RStudio.^21^ Importantly, WHO ROs also conducted similar processes on a weekly basis to independently validate data collected at their level, with results being shared between WHO HQ and ROs to ensure consistency.

Data for hospitalisations and ICU admissions were validated by two epidemiologists through the visual inspection of an internal Microsoft Power BI^22^ dashboard used to monitor reporting completeness and detect anomalies at regional and country levels. The validation process involved identifying outliers and anomalies, including extreme increases in the number of hospitalisations and ICU admissions, higher numbers of hospitalisations than reported cases and higher numbers of ICU admissions than hospitalisations. Whenever discrepancies arose in the number of cases, deaths, hospitalisations or ICU admissions, the team at WHO HQ sought clarification from the relevant ROs.

Following data validation, distinct R scripts were executed separately by two data analysts and one geographical information systems specialist for the production of standardised narratives, epidemic curves, maps and other graphs relating to global and regional trends of COVID-19 cases, deaths, hospitalisations and ICU admissions.

### Drafting, review and dissemination of the WEU

While the WEU’s global and regional overviews featured mostly automated narratives, the narratives of the other sections were drafted manually by different subject-matter experts, including epidemiologists, clinicians, immunologists, virologists and others. All draft narratives and figures were incorporated by an information manager using Microsoft Word.^23^

Drafts then went through two rigorous rounds of internal peer review by a core group of 6–8 epidemiologists, infectious disease experts and communication specialists to ensure that the presented information was scientific and clear. When the revisions were completed, final clearance for publication was provided by WHO’s COVID-19 Technical Lead and the WEU was published by a health information manager on a dedicated WHO website and disseminated to external partners via email.

## Challenges

Despite the existence of standardised procedures for the production of information products for acute events such as influenza and Ebola virus disease, the development of the WEU was complex given the scale and urgency of the COVID-19 pandemic, the breadth and depth of the collected data and the pressing demand for real-time analysis of the epidemiological situation. The infrastructure for generating the WEU was designed and regularly refined while the report was simultaneously being published on a weekly basis. The automation of certain processes, such as the collection of reported cases and deaths or the generation of graphs and maps, allowed us to optimise the use of staff time, while increasing consistency and accuracy. Nevertheless, many components of the WEU continued to require significant manual effort and meticulous scrutiny. Therefore, the production process was highly demanding and resource intensive.

In its attempt to provide a holistic overview of the pandemic, the WEU exemplified many obstacles inherent to the global surveillance of the COVID-19 pandemic. For example, during the course of the pandemic, different countries and regions had varying levels of data accessibility and geographic representativeness, with some areas persistently experiencing incomplete or delayed reporting. These discrepancies and delays were inevitably reflected in the figures presented in the WEU, limiting their utility to assess the true spread and impact of the virus. Furthermore, very limited reporting of hospitalisation data made it difficult to draw firm conclusions regarding hospitalisation and infection trends as well as hospital and ICU capacity. Timely sharing of genomic sequencing data from countries was also challenging due to the median lag between the collection and submission of SARS-CoV-2 sequences to GISAID varying significantly across countries, ranging from approximately 2 weeks to over 9 months.^24^ Such a delay affected the confidence in the analyses of SARS-CoV-2 variants reported in the WEU. Additionally, countries used different case, death and hospitalisation definitions; age categories; testing practices;and reporting protocols, despite the available WHO guidance.^11^ These differences hindered data harmonisation, influencing the comparability and interpretation of the information provided in the WEU. Furthermore, the data included in the WEU were predominantly based on reported cases and deaths, which by definition did not capture the true burden of the COVID-19 pandemic. Under-reporting and differences in access to healthcare as well as healthcare-seeking behaviour across populations also significantly affected estimations of the morbidity and mortality associated with COVID-19.

The above-mentioned challenges are now being exacerbated by the ongoing deprioritisation, defunding and scaling down of SARS-CoV-2 surveillance activities, alongside the limited capacity to track rapid diagnostic tests and the persistent declines in the use of reverse-transcription polymerase chain reaction (RT-PCR) for testing. WHO recognises these challenges and has published policy briefs^25 26^ as well as the WHO Director General’s standing recommendations^27^ for sustaining national capacity gains relating to COVID-19 and other infectious diseases. Failure to address these challenges hampers our collective ability to effectively detect, evaluate, and monitor current and future variants as well as the morbidity and mortality linked to them.

## Future directions and recommendations

COVID-19 data shared by Ministries of Health and public health agencies with WHO were and still are vital not only for the WEU but for all aspects of the global COVID-19 response. Moving forward, as recommended in WHO’s COVID-19 Strategic Preparedness and Response Plan for April 2023–April 2025,^28^ it is critical that countries maintain core SARS-CoV-2 surveillance capacities and activities to inform ongoing public health measures and ensure that future surges of COVID-19 are rapidly detected, allowing for swift action to prevent larger outbreaks.

Specifically, countries should continue to make efforts to improve systems for the timely collection, quality assurance and reporting of data. This can be achieved by sustaining investments in national surveillance systems, integrating disease surveillance systems where relevant including into sentinel surveillance systems, maintaining genomic surveillance, expanding wastewater surveillance, harmonising data standards and encouraging prompt and thorough reporting of data. This can entail leveraging existing mechanisms or devising new tools for facilitating the sharing of information as well as expertise. To enhance the efficiency and timeliness of reporting, countries should strive to automate the generation of their information products fully or at least partially. Among epidemiological indicators, priority should be given to the collection and reporting of data on deaths, hospitalisations and ICU admissions. Furthermore, clear definitions of each indicator should be provided to ensure clarity in communication, reduce the risk of misinterpretation, and facilitate data analysis.

As previously recommended by WHO,^25^ countries should perform SARS-CoV-2 testing strategically and incorporate where possible and feasible into existing (particularly respiratory) disease surveillance mechanisms, such as the Global Coronavirus Laboratory Network,^28^ the Global Influenza Surveillance and Response System,^29^ and the Severe Acute Respiratory Infections network.^30^ Countries should also continue to strengthen genomic surveillance for SARS-CoV-2 and other pathogens with epidemic and pandemic potential, regularly sharing up-to-date genomic as well as associated epidemiological and clinical data, to allow for an understanding of the phenotypic characteristics of emerging variants; sharing can be done through official international reporting systems and/or publicly available platforms.

Finally, in line with WHO’s Health Emergency Preparedness, Response and Resilience strategy,^31^ countries should work towards implementing collaborative surveillance, which is the coordinated effort among health and non-health stakeholders to integrate various types of data and approaches to holistically inform public health action. This involves triangulating and synthesising information from environmental, event-based, participatory, sentinel and seroepidemiological surveillance, among others.

## Conclusion

Thanks to the collaborative efforts of national health authorities around the world and WHO’s dedication to accuracy and transparency, the WEU provided the international community with comprehensive and timely analyses of the global COVID-19 situation. Its global readership is a testament to the WEU’s extensive impact and reach. The publication’s relevance goes beyond COVID-19 as the WEU offers a blueprint for the development of information products that can support the response to future major health emergencies.

The experience of the WEU underscores the importance of having regular reports to inform the public, global actors and response operations during acute health events. The WEU has also shown that, while automation and artificial intelligence can facilitate processes such as data collection, analysis and information synthesis, human interpretation by subject-matter experts remains crucial to ensure quality control, provide contextual understanding and translate raw data into actionable insights.

While the progress in the global COVID-19 surveillance has been commendable, several challenges persist, affecting our ability to detect and respond to new waves of infection. Addressing these challenges requires sustained international cooperation, a renewed commitment to science and public health, predictable funding and investments in technological innovation. As we transition out of the acute phase of the pandemic, WHO will continue to provide evidence-based information on COVID-19, improve existing processes for data collection and information sharing, and support all countries to address every aspect of the COVID-19 response and recovery.

## Data Availability

Data are available in a public, open access repository. Data are available on reasonable request. All data reported on the WHO’s COVID-19 Weekly Epidemiological Update (WEU) are publicly available. Data relating to the webpage that hosted the WEU are available on reasonable request.
