# Supplementary material for: Informing the pandemic response: the role of the WHO’s COVID-19 Weekly Epidemiological Update
Source: BMJ Glob Health. 2024 Apr 4;9(4):e014466. doi: 10.1136/bmjgh-2023-014466 (PMC11002403; doi:10.1136/bmjgh-2023-014466)
Supplement: Supplementary data [file bmjgh-2023-014466supp001.pdf]

Appendix 1. COVID-19 WEU Special Focus: Topics covered and dates of publication.

Date of publication	Special Focus
19 January 2021	Children, COVID-19, and transmission in schools
27 January 2021	Solidarity II forum and use of international standards for seroepidemiology surveys
2 February 2021	COVID-19 and health workers
9 February 2021	How COVAX is distributing the first COVID-19 vaccines to prioritized countries in all six WHO regions
16 February 2021	The global influenza surveillance and response system
23 February 2021	WHO COVID-19 vaccine policy recommendations
2 March 2021	1. COVID-19 trade, travel and points of entry<br>2. The importance of fit, filtration and breathability of non-medical (fabric) masks in the context of COVID-19
9 March 2021	1. Global Influenza Surveillance and Response System – best practices for integrating influenza and COVID-19 sentinel surveillance<br>2. SARS-CoV-2 sero-epidemiology in Kenya
16 March 2021	Building and maintaining trust - what countries should do to prepare communities for a COVID-19 vaccine, treatment, or a new test; and
23 March 2021	Release of WHO COVID-19 Detailed Surveillance Dashboard
30 March 2021	COVID-19 and Health and Care Workers (HCWs)
20 April 2021	1.WHO COVID-19 global rapid risk assessment<br>2.Pandemic influenza surveillance—drawing a parallel with the COVID-19 pandemic
4 May 2021	1.World Hand Hygiene Day, 5 May 2021<br>2.WHO partnership with SeroTracker — synthesizing “real-time” seroprevalence data to support global pandemic response
1 June 2021	Lessons learned during the early phases of rolling out COVID-19 vaccines, with a particular focus on low-and-middle income countries (LMICs).
15 June 2021	Update about strengthening public health intelligence through event-based surveillance, specifically learning from the COVID-19 pandemic.
22 June 2021	Global Consultation on SARS-CoV-2 Variants of Concern and their Impact on Public Health Interventions.
29 June 2021	Overview of current challenges in the context of the COVID-19 pandemic, as well as a summary of WHO global conference on communicating science during health emergencies.
6 July 2021	Variant working definitions, as well as other variants and amino acid changes under monitoring
13 July 2021	A synopsis of the latest WHO COVID-19 Rapid Risk Assessment, which aims to review the current status of global public health risks associated with the pandemic through an in-depth hazard, exposure and context assessment
20 July 2021	The release of a WHO COVID-19 detailed surveillance data dashboard, including a downloadable database feature
27 July 2021	The evaluation of COVID-19 vaccines and their effectiveness in real world settings.
17 August 2021	COVID-19 in Prisons
14 September 2021	COVID-19 in children and adolescents

28 September 2021	Approaches to determining waning COVID-19 vaccine effectiveness
26 October 2021	1.WHO COVID-19 global rapid risk assessment<br>2.Age and sex distribution from WHO COVID-19 global surveillance
23 November 2021	Points of entry, international travel and transport in the context of the COVID-19 pandemic
18 January 2022	WHO COVID-19 global rapid risk assessment
15 March 2022	Contact tracing and quarantine in the context of the Omicron SARS-CoV-2 variant: interim guidance
12 April 2022	Update on WHO COVID-19 global rapid risk assessment
20 April 2022	Applying Geographic Information Systems to the COVID-19 response
18 May 2022	Environmental surveillance for SARS-CoV-2 to complement public health surveillance
15 June 2022	Mass gathering events during disease outbreaks and WHO's recommended risk-based approach to decision-making for mass gathering events
29 June 2022	Relative vaccine effectiveness
13 July 2022	WHO global situational alert system
16 March 2023	COVID-19 vaccination status of health and care workers
13 April 2023	Updated Interim Guidance on Adjusting Public Health and Social Measures for COVID-19
